# Senescent mesenchymal stem cells promote colorectal cancer cells growth via galectin-3 expression

**DOI:** 10.1186/s13578-015-0012-3

**Published:** 2015-05-06

**Authors:** Yanju Li, Xiao Xu, Lihua Wang, Guangjin Liu, Yanqi Li, Xiaobing Wu, Yongguang Jing, Haiyan Li, Guihua Wang

**Affiliations:** Department of Hematology, Affiliated Hospital of Guiyang Medical College, No. 28, Guiyi Street, Yunyan District, Guiyang, Guizhou Province 550004 China; Skin Regeneration Department of General Hospital of Chinese Armed Police, No. 69 Yongding Road, Haidian District, Beijing, 100039 China; Department of Hematology, The Second Hospital of Hebei Medical University, Hebei Key Laboratory of Hematology, Hebei Institution of Hematology, 215 West Heping Road, Shijiazhuang City, Hebei Province 050000 China; Yanzhou Hospital of Traditional Chinese Medicine, No. 43 Dongqiao North Road, Yanzhou District, Jining, Shandong Province 272100 China

**Keywords:** Cellular senescence, Mesenchymal stem cells, Galectin-3, LoVo cells

## Abstract

**Background:**

Cellular senescence is linked to aging and tumorigenesis. The senescence of mesenchymal stem cells (MSCs) may influence the tumor growth, metastasis, and angiogenesis by secreting a variety of cytokines and growth factors.

**Results:**

The conditioned media of adipose derived MSCs (AD-MSCs) stimulated the proliferation of human LoVo colorectal-cancer cells, and the replicative senescent MSCs had the more obvious effects in comparison to that of premature AD-MSCs. Analysis of the factors secreted in the MSCs culture media determined that senescent MSCs expressed and secreted high levels of galectin-3. Galectin-3 expression correlated with the stimulatory effect of senescent AD-MSCs on LoVo cells proliferation, as knockdown of galectin-3 in senescent AD-MSCs significantly reversed the effect of MSCs–mediated growth stimulation of LoVo cells. Furthermore, the simultaneous addition of recombinant galectin-3 to the co-culture systems partially restored the tumor-promoting effect of the senescent AD-MSCs. Analysis of the mechanisms of senescent MSCs and galectin-3 on LoVo cells signal transduction determined that senescent MSCs and exogenous galectin-3 promoted cell growth by activating the mitogen-activated protein kinase (MAPK) (extracellular signal-regulated kinase [ERK]1/2) pathway.

**Conclusions:**

Senescent MSCs may alter the tissue microenvironment and affect nearby malignant cells via cytokine secretion, and galectin-3 is an important mediator of senescent AD-MSC–mediated stimulation of colon cancer cell growth. Therefore, thorough assessment of AD-MSCs prior to their implementation in clinical practice is warranted.

## Background

Cellular senescence, a state of irreversible growth arrest, can be triggered by many mechanisms, including telomere shortening, epigenetic derepression of the cyclin-dependent kinase inhibitor 2A/ADP ribosylation factor (INK4a/ARF) locus, and DNA damage [1]. Cellular senescence is linked to aging and tumorigenesis, and has been proposed as a suppressive mechanism against the development of cancer [2, 3]. However, recent studies have revealed that cellular senescence also has tumor-promoting effects, and the accumulation of senescent cells during the aging process in vivo may contribute to the age-related increase in cancer incidence [4].

Mesenchymal stem cells (MSC), which possess broad and potent multilineage differentiation potential and potent immunoregulatory properties, are the optimal source for stem cell transplant in clinical applications [5, 6]. Similar to other adult somatic cells, long-term culture in vitro leads to MSC senescence, which results in growth arrest and reduced differentiation [7, 8]. The replicative senescence of MSCs is evinced by telomere shortening, enlargement and flattening of the characteristic stem cell morphology, reduced proliferation rate, and altered secretory profile [8, 9]. The function of adult tissue-specific stem cells declines with age, and aged MSCs could be more deleterious as they can greatly impair tissue homeostasis and repair [9, 10]. MSCs from aged patients with coronary artery disease have impaired angiogenic potential and reduced proangiogenic factor secretion [11]. Senescent MSCs may contribute to the physiological decline in tissue homeostasis and to the increased risk of neoplasm during aging [9].

Recently report showed that MSCs stimulated invasion, survival and tumorigenesis of colorectal cancer cells through the release of soluble NRG1, activating the HER2/HER3-dependent PI3K/AKT signalling cascade in colorectal cancer cells [12]. In this study, we showed that replicative senescent AD-MSCs significantly promoted the proliferation of LoVo colorectal-cancer cells in comparison to premature AD-MSCs, and the expression of galectin-3, a powerful modulator of cell migration and spread in carcinoma cells, correlated with the stimulatory effect of senescent AD-MSCs on colorectal cancer cells proliferation. Therefore, thorough assessment of AD-MSCs prior to their implementation in clinical practice is warranted.

## Results

### Characterization of P30-MSCs

**Fig. 1:**
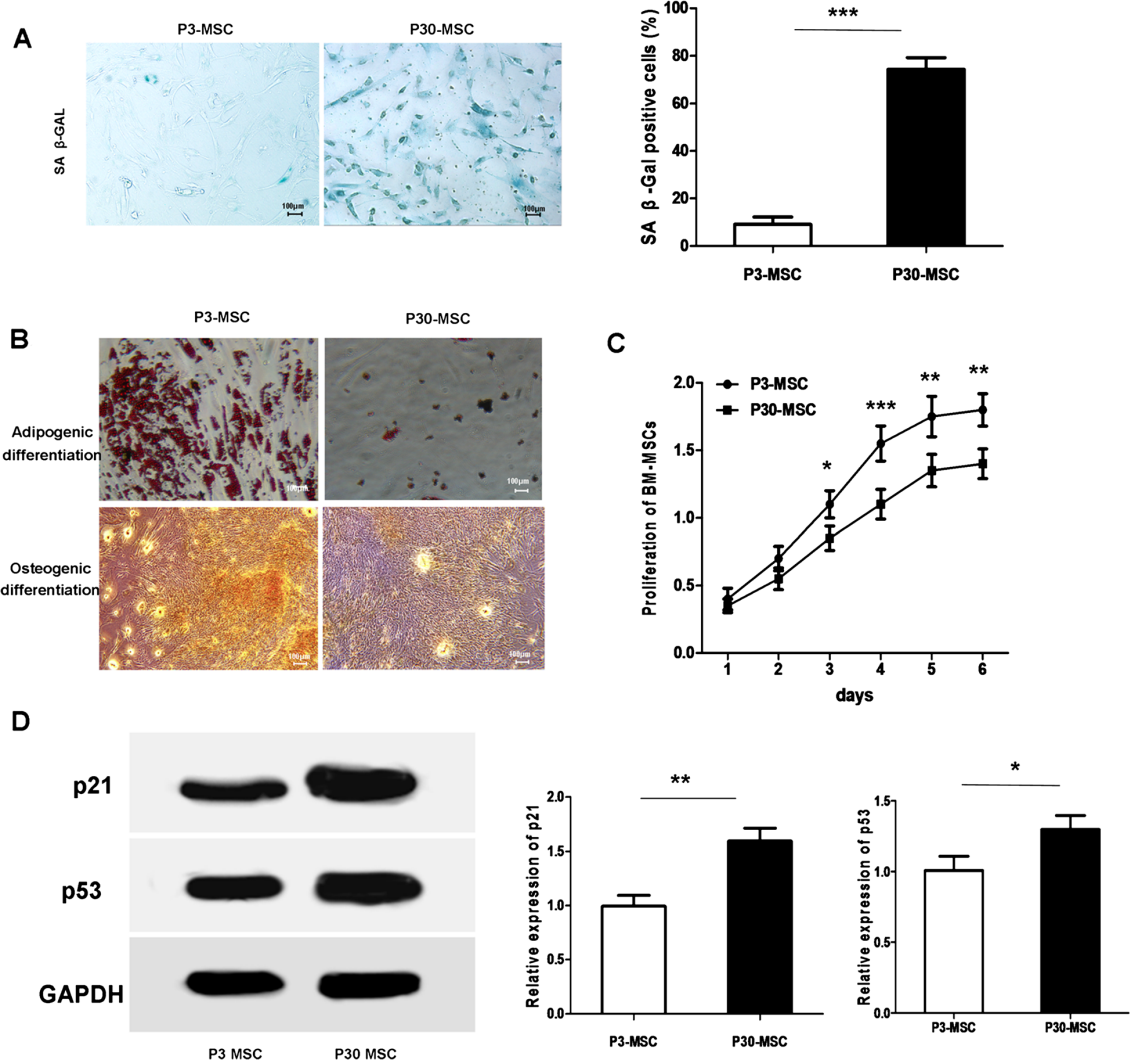
Characterization of senescent AD-MSCs. (**a**) SA-β-Gal staining of senescent MSCs (P30 AD-MSCs) or pre-senescent MSCs (P3 AD-MSCs). (**b**) Oil Red O and Alizarin red staining of differentiated AD-MSCs. (**c**) The proliferation potential of AD-MSCs by CCK-8 analysis. (**d**) Western blot analysis of AD-MSC P21 and P53 expression. GAPDH, glyceraldehyde-3-phosphate dehydrogenase internal control

In the first few generations, the AD-MSCs exhibited fibroblast-like morphology. After 30 passages, the MSCs appeared longer and larger, with accumulation of granular cytoplasmic inclusions. The percentage of positive SA-β-Gal staining was increased significantly in the replicative P30-MSCs (Fig. 1a). The AD-MSCs were also capable of osteogenic and adipogenic differentiation when cultured in the appropriate inducing media. Assessment of the degree of osteogenic and adipogenic differentiation via Alizarin Red S and Oil Red O staining, respectively, revealed a sharp decline in the adipogenic and osteogenic potential of P30-MSCs compared to P3-MSCs (Fig. 1b). CCK-8 analysis showed that the proliferation potential of AD-MSCs declined significantly with cell replicative passaging (Fig. 1c). Western blot analysis of p53 and p21 expression in the AD-MSCs showed that p53 and p21 expression increased gradually with passage (Fig. 1d). Flow cytometric analysis demonstrated that both P3-MSCs and P30-MSCs were positive for CD73, CD90, and CD105, and negative for CD34, CD45, and HLA-DR (data not shown).

### CM-P30 promoted colon cancer cell proliferation

**Fig. 2:**
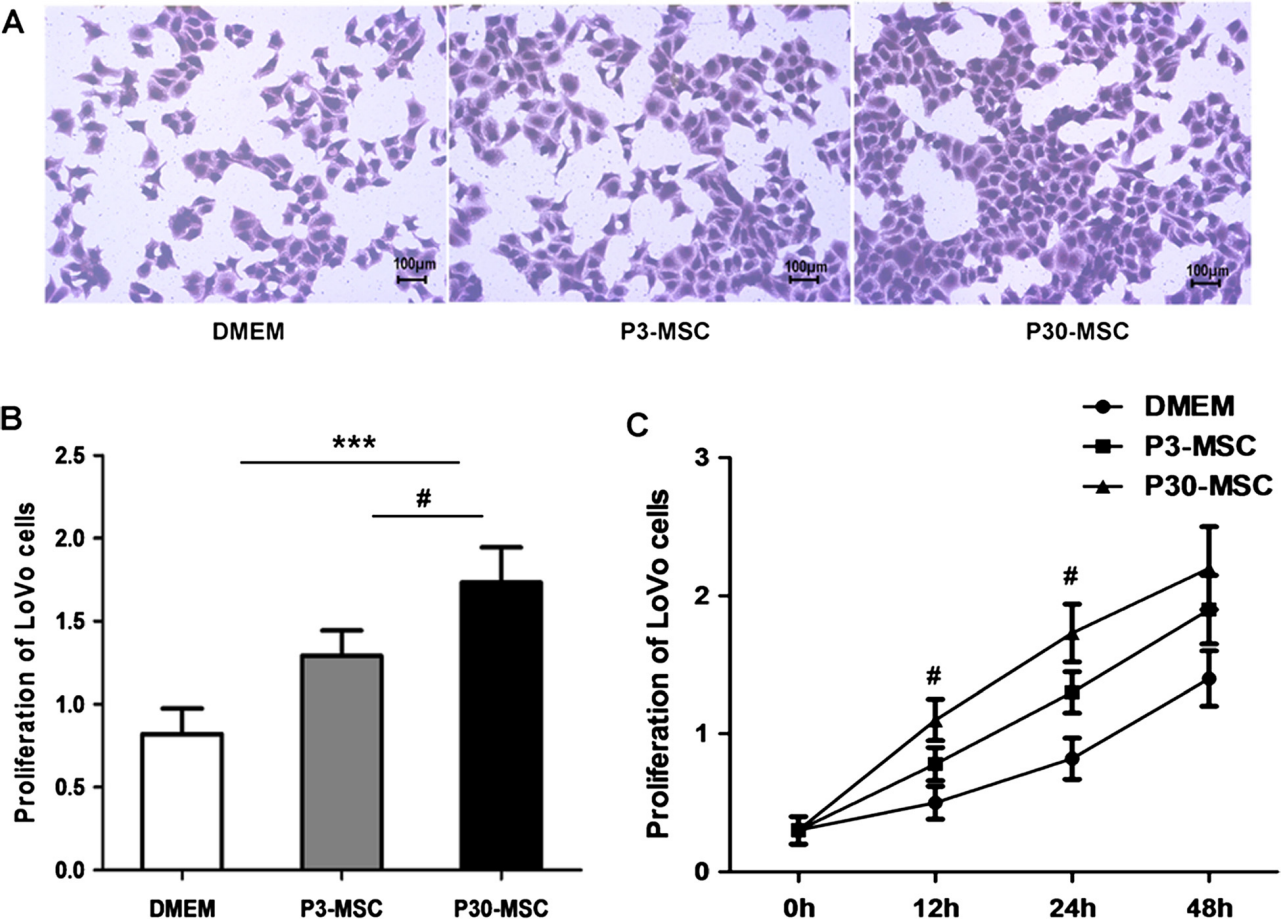
The CM of AD-MSCs promotes LoVo cell proliferation in vitro. (**a**) Morphology of LoVo cells incubated with the CM of AD-MSCs for 24 h. (**b**) The proliferation assays of LoVo cell with CM of AD-MSCs for 24 h. (**c**) The proliferation assays of LoVo cell with CM of AD-MSCs for 12 h, 24 h and 48 h. All experiments were repeated at least three times, normal DMEM without cell medium (DMEM) was used as the control. ***P < 0.001 versus control; ^#^ *P* < 0.05, P3-MSC versus P30-MSC

To determine the effect of P30-MSCs on colon cancer cell growth, LoVo cells were cultured with concentrated CM-P3 or CM-P30 for 48 h. As shown in Fig. 2a-c, the conditioned medium of MSCs can promoted LoVo cell proliferation, and the LoVo cells cultured with CM-P30 spread and grew faster than cells cultured with culture medium alone or with CM-P3. However if the cells were incubated for 48 h, both of CM-P3 and CM-P30 treated colon cancer cell groups achieved similar proliferation levels.

### Galectin-3 contributed to P30-MSC stimulation of colon cancer cell growth

**Fig. 3:**
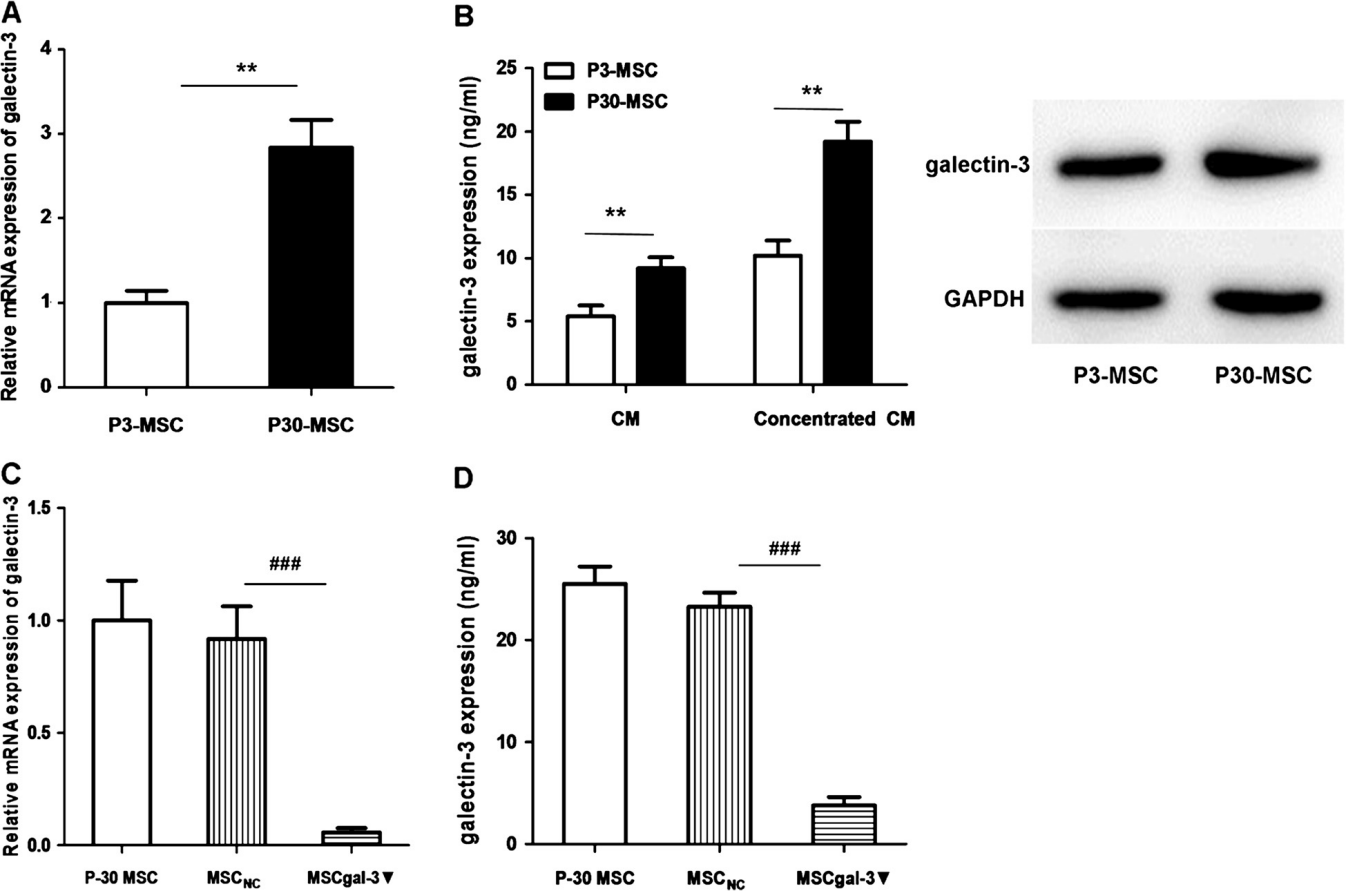
Galectin-3 expression in P3-MSC or P30-MSC. (**a**) *LGALS3* mRNA expression in P3-MSCs or P30-MSCs. (**b**) Galectin-3 protein levels in the CM of AD-MSCs. (**c**) Decreased expression of *LGALS3* mRNA in P30-MSCs following treatment with galectin-3 siRNA. (**d**) Decreased expression of galectin-3 protein in CM-P30 following treatment with galectin-3 siRNA. MSC_gal-3▼_, MSCs treated with galectin-3 siRNA; MSC_NC_, MSCs treated with negative control of siRNA. All experiments were repeated at least three times. **P < 0.01, P3-MSC versus P30-MSC; ^###^ *P* < 0.001, MSC_NC_ versus MSC_gal-3▼_

Accumulated evidence indicates that galectin-3 is closely involved in tumor cell proliferation, transformation, migration, invasion, and metastasis [13]. We analyzed galectin-3 expression in CM-P3 or CM-P30 with quantitative PCR (Q-PCR) and ELISA, and found that both mRNA and protein levels of galectin-3 were significantly upregulated in the AD-MSCs during senescence (Fig. 3a-b), suggesting that galectin-3 may have been involved in the P30-MSC–mediated growth stimulation of LoVo cells.

To examine whether galectin-3 secretion in P30-MSCs stimulates LoVo cell proliferation, we blocked galectin-3 expression in P30-MSCs with a galectin-3–specific siRNA. Q-PCR and ELISA data showed that *LGALS3* mRNA expression was decreased and that galectin-3 secretion in CM-P30 was significantly reduced following siRNA treatment (Fig. 3c, d).

**Fig. 4:**
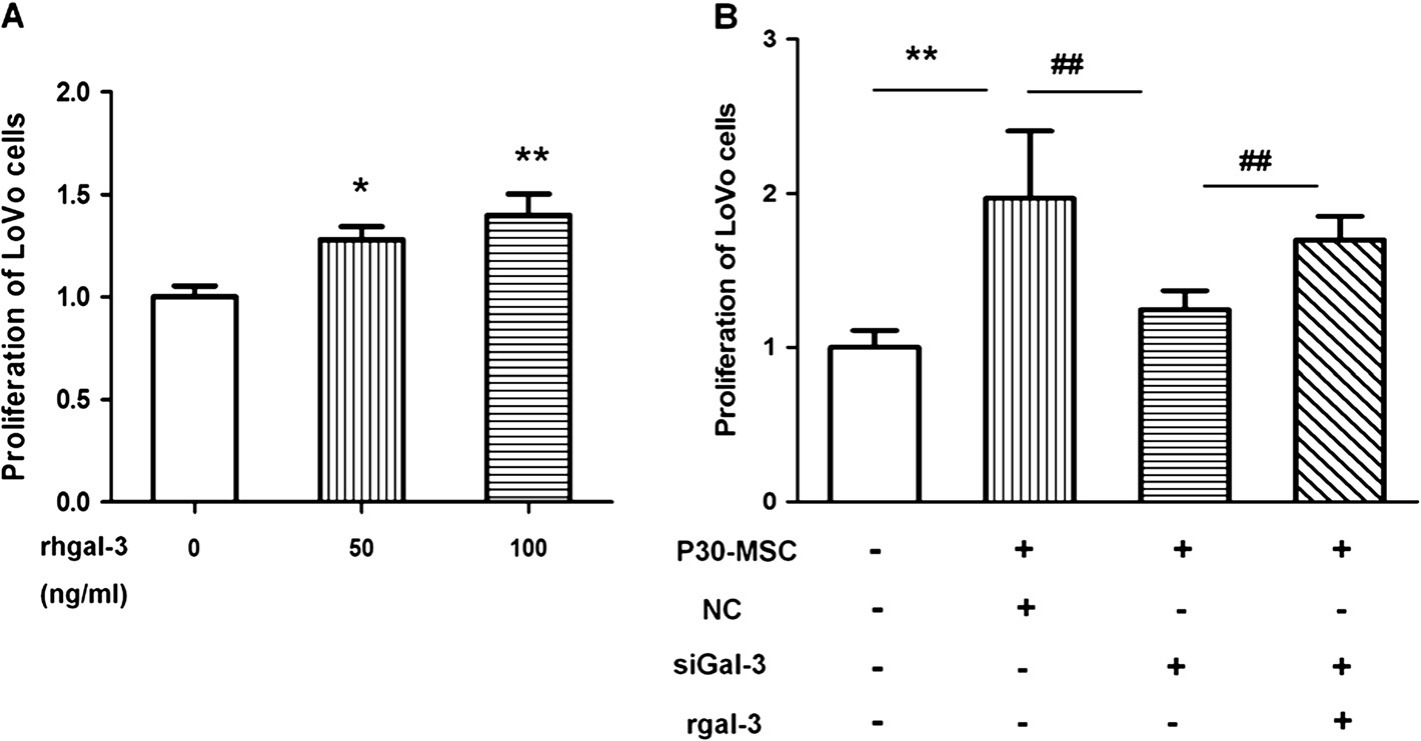
Galectin-3 is an important mediator of P30-MSC–mediated stimulation of LoVo cell growth. (**a**) Proliferation of LoVo cells incubated with 50 or 100 ng/ml recombinant galectin-3. (**b**) Proliferation of LoVo cells incubated with CM of P30-MSCs, which treated with galectin-3 siRNA or NC. Control, normal DMEM without cell medium; siGal-3, galectin-3 siRNA; rgalectin-3, recombinant galectin-3. All experiments were repeated at least three times. *P < 0.05,**P < 0.01 versus control; ^##^ *P* < 0.01, versus siGAL-3

LoVo cells were incubated with 50 or 100 ng/ml recombinant galectin-3 for 24 hours, and the proliferation of LoVo cells were evaluated by CCK-8. As Fig. 4a showed that the rgalectin-3 enhanced the growth of LoVo cells. LoVo cells were then incubated with CM-P30 pre-treated with the galectin-3 siRNA or NC, and the knockdown of galectin-3 in senescent AD-MSCs significantly reversed the effect of MSCs–mediated growth stimulation of LoVo cells (Fig. 4b). Furthermore, the simultaneous addition of 100 ng/ml recombinant galectin-3 to the co-culture systems partially restored the tumor-promoting effect of the senescent AD-MSCs.

### P30-MSCs promoted ERK1/2 activation in colon cancer cells

**Fig. 5:**
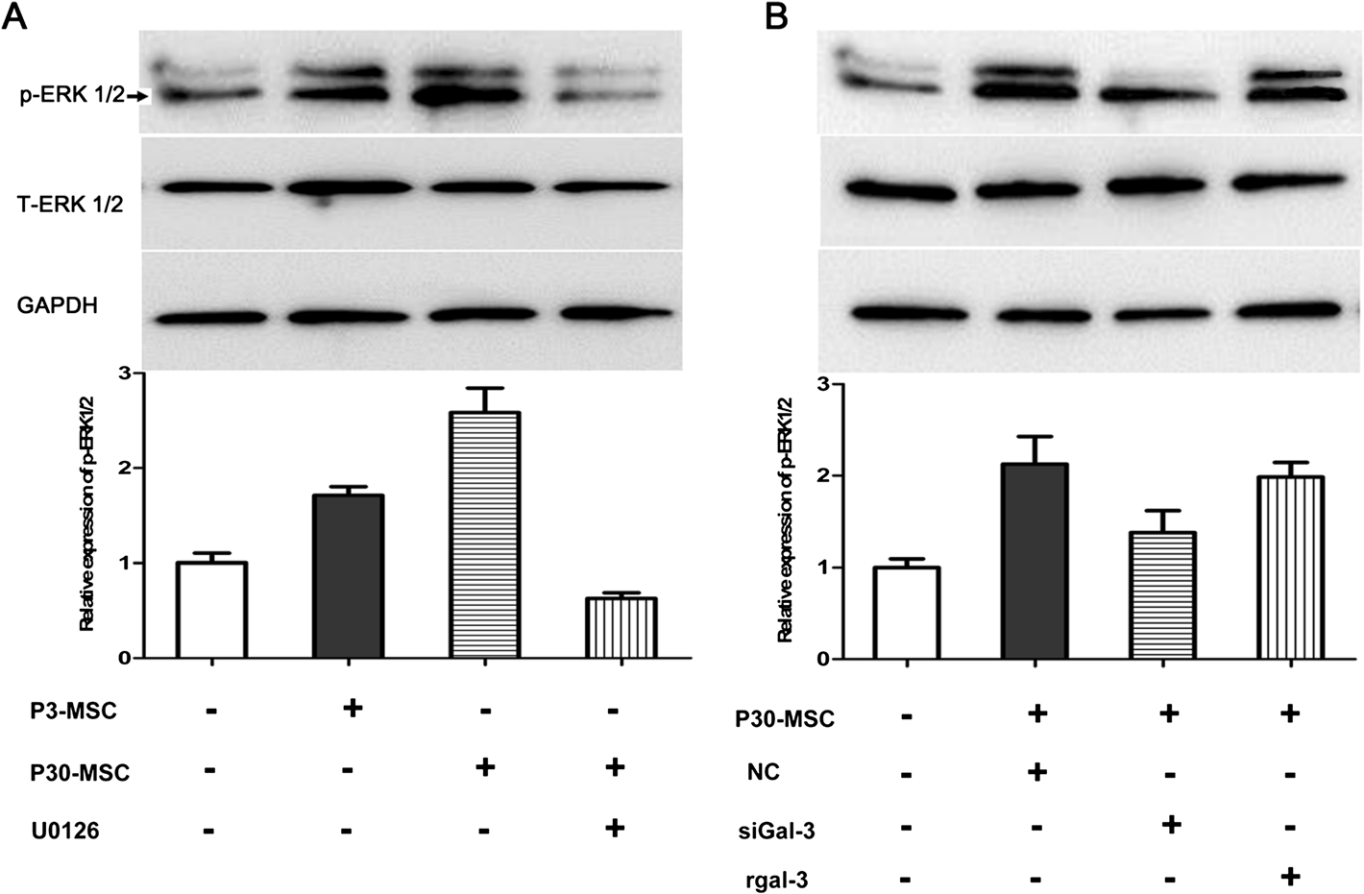
Western blot analysis of P30-MSC and exogenous galectin-3 promotion of ERK1/2 activation in LoVo cells. (**a**) the CM of MSCs promoted ERK1/2 phosphorylation in the LoVo cells, which were aborted by U0126 for 60 min treatment. (**b**) LoVo cells incubated with CM of P30-MSCs, which treated with or without galectin-3 siRNA.siGal-3, galectin-3 siRNA; rgalectin-3, recombinant galectin-3; GAPDH was used as the internal control. All experiments were repeated at least three times. ^##^ *P* < 0.01 versus control; **P < 0.01 versus siGAL-3

As reported previously [14], exogenous galectin-3 induces the extracellular signal-regulated kinases (ERK1/2) phosphorylation in cancer cells, and the activation of ERK1/2 are associated with cancer cell proliferation and survival [15, 16]. Our western blot data were showed in Fig. 5a, the CM of MSCs promoted ERK1/2 phosphorylation in the LoVo cells and that CM-P30 had a greater stimulative effect on ERK1/2 activation. Moreover, the phosphorylation of ERK1/2 induced by CM-P30 of MSCs were aborted by U0126, the specific inhibitor of MEK1/2, suggesting that the signal was transferred through a specific Raf-MEK1/2-ERK1/2 pathway to activate ERK1/2. We then knocked down galectin-3 expression in the P30-MSCs and compared the promoter effect of the CM-P30 on ERK1/2 phosphorylation to that of the CM of MSCs treated with MSC_NC_. Galectin-3 knockdown diminished the CM-P30–induced ERK1/2 phosphorylation; however, the addition of exogenous galectin-3 to the CM restored ERK1/2 activation in the LoVo cells (Fig. 5b).

## Discussion

Recent studies have shown that a pool of molecules secreted by senescent cells, referred to as having the senescence-associated secretory phenotype (SASP), is associated with arrest of cell proliferation and may contribute to it via the autocrine/paracrine pathways [10, 17]. Our data revealed that the MSCs had the typical senescence-associated characteristics and SASP after repeated passage, marked by the appearance of senescence-associated morphological features, decreased proliferation, SA-β-Gal positivity, induced p53 and p21 expression, and increased galectin-3 expression. We then showed that CM-P30 promoted colon cancer cell proliferation. In the co-culture experiments, we demonstrated that galectin-3 mediated the promoter effects of AD-MSCs on colon cancer cell proliferation to some extent, as specific knockdown of galectin-3 with siRNA significantly reversed the MSC-mediated stimulation of colon cancer cell growth.

The tumor microenvironment is increasingly regarded as an important regulator of malignant progression of cancer cells [18]. MSCs may secrete a variety of cytokines and growth factors that influence tumor growth, metastasis, and angiogenesis [19, 20]. Karnoub et al. [19] showed that MSCs play a pivotal role in colon cancer progression and metastasis; when recruited into breast cancer stroma, bone marrow MSCs tended to facilitate breast cancer cell metastasis and regulate cancer stem cell behavior via the secretion of the chemokine CCL5. Recent studies have shown that the CM of MSCs enhances tumor growth, indicating that the factors secreted by MSCs have profound effects on “reprogramming” tumor growth [18, 21], and the senescent umbilical cord MSCs promoted the proliferation and migration of breast cancer cells [22].

Of the MSC SASP factors, we found that galectin-3 was an important mediator of cancer-promoting activity. A unique chimera-type member of the β-galactoside–binding soluble lectin family with a molecular mass of 29–35 kD, galectin-3 is implicated in a variety of biological functions [23, 24]. Previously, Liu et al. showed that galectin-3 is secreted into the CM of UC-MSCs and is involved in the immunosuppressive function of UC-MSCs [25]. In addition, galectin-3 expression in most cancer cells is increased, and is associated with growth and metastases in pancreatic and breast cancer systems [26]. A recent report [27] showed that galectin-3 is involved in the nicotine-induced promotion of apoptosis resistance of breast cancer cells and that it promotes cancer cell growth and protects cells from apoptosis induced by chemotherapeutic drugs. Baptiste et al. [28] showed that galectin-3 was a powerful modulator of cell adhesion and spread in breast carcinoma cells and that the exogenous addition of recombinant galectin-3 promoted the growth of galectin-3–null cells. However, our data showed that recombinant galectin-3 has a weaker effect on cancer cell growth than senescent MSCs, suggesting that other cytokines secreted from senescent MSCs may be associated with the stimulation of colon cancer cell growth.

Lastly, we analyzed the mechanisms of senescent MSCs and galectin-3 on colon cancer cell signal transduction, finding that senescent MSCs and exogenous galectin-3 promoted colon cancer cell growth by activating the MAPK (ERK1/2) pathway. The extracellular signal-regulated kinases (ERK1/2) which are serine/threonine protein kinases, as one of the most important regulator of key cellular processes [15], involved in carcinogenesis due to their ability to stimulate cell proliferation and survival [16]. The MAPK pathways are activated by diverse extracellular and intracellular stimuli including peptide growth factors, cytokines, hormones, and various cellular stressors [29]. Gao et al. [14] showed that exogenous galectin-3 induces ERK1/2 phosphorylation in cancer cells, and our results are consistent with this reports.

In summary, our study suggests that senescent MSCs may alter the tissue microenvironment and affect nearby malignant cells via cytokine secretion, and that galectin-3 is an important mediator of senescent AD-MSC–mediated stimulation of colon cancer cell growth. Therefore, thorough assessment of AD-MSCs prior to their implementation in clinical practice is warranted.

## Materials and methods

### Isolation and preparation of senescent AD-MSCs

The AD-MSCs were isolated and obtained by density gradient centrifugation, approved by the Ethics Committee of the Affiliated Hospital of Guiyang Medical College. Briefly, AD-MSCs were isolated and cultured according to a collagenase digestion protocol. Mononuclear cells were plated in α-minimum essential medium (Invitrogen, Carlsbad, CA, USA) containing 10 % fetal bovine serum (FBS) (HyClone, Utah, USA), and cultured in a humidified incubator containing 5 % CO2 at 37 °C.

AD-MSCs were further cultivated to confluence and were amplified, and replicative senescent cells were passaged serially over 30 passages when the typical senescence-associated morphological features appeared. Passage 3 (P3) and P30 cells were referred to as “young” (P3-MSCs) and “senescent” MSCs (P30-MSCs), respectively.

### Characterization of senescent AD-MSCs

Senescence-associated β-galactosidase (SA-β-Gal) staining was performed using an SA-β-Gal staining kit (Cell Signaling Technology, Beverly, MA, USA) according to the manufacturer’s instructions. For the proliferation analysis, MSCs were counted every 24 hours for 5 days using a cell counting kit-8 (CCK-8) (DoJinDo, ShangHai, China). All experiments were performed three times.

AD-MSCs surface marker analysis by flow cytometry was performed on a FACSCalibur unit (Becton Dickinson Biosciences, San Jose, CA, USA); cells were stained with phycoerythrin-conjugated antibodies against CD105, CD73, CD90, CD45 and HLA-DR (Becton Dickinson Biosciences). For osteogenic and adipogenic differentiation, MSCs were incubated with MesenCult Osteogenic or Adipogenic Stimulatory Medium (STEMCELL Technologies, Vancouver, Canada) for 2–3 weeks. Osteogenic and adipogenic differentiation was evaluated using Alizarin Red S and Oil Red O (Sigma-Aldrich, St. Louis, MO, USA) staining, respectively.

p21 and p53 protein levels were analyzed by western blotting. Proteins were extracted from 1 × 10^6^ P3-MSCs or P30-MSCs with 1 ml radioimmunoprecipitation assay (RIPA) buffer (Sigma-Aldrich), separated by 10 % sodium dodecyl sulfate–polyacrylamide gel electrophoresis (SDS-PAGE), and transferred to Immobilon polyvinyl difluoride (PVDF) membranes. The membranes were incubated with antibody against p21 and p53 (Abcam, Cambridge, MA, USA), followed by goat anti-rabbit horseradish peroxidase (HRP)-conjugated antibody (Santa Cruz Biotechnology, Santa Cruz, CA, USA), and visualized with SuperSignal West Pico Chemiluminescent Substrate (Millipore, Billerica, MA, USA).

### Preparation of conditioned medium

To harvest the MSC conditioned medium (CM), 80 % confluent P3-MSC and P30-MSC cultures were extensively washed with phosphate-buffered saline (PBS) and incubated in serum-free culture medium for 24 h. Then, the CM from the P3-MSCs (CM-P3) and P30-MSCs (CM-P30) was collected and concentrated to 1/2× volume with an ultra-filtration membrane with a molecular weight cut-off of 3 kD by centrifuging at 5000 rpm for 30 min at 4 °C. The concentrated CM was then filtered through a 0.22-μm membrane and stored at −80 °C until used.

### Cell proliferation assay of colon cancer cells

The human LoVo colon cancer cell line was obtained from the American Type Culture Collection (ATCC). LoVo cells were established from a metastatic nodule resected from a 56-year-old Caucasian male colorectal adenocarcinoma patient [30].

The LoVo cells were cultured in phenol red-free Dulbecco’s modified Eagle’s medium (DMEM) (Invitrogen, Carlsbad, CA, USA) supplemented with 10 % FBS; 2.5 × 10^3^ cells/well were plated in 200μl growth medium in 96-well plates. After 24 h, the medium was replaced with concentrated conditioned medium of CM-P3 and CM-P30 as for 12h, 24 h or 48 h. Tumor cell proliferation was evaluated using Cell Counting Kit-8 according to the manufacturer’s instructions.

### Analysis of galectin-3 expression in MSCs

We analyzed galectin-3 (*LGALS3*) mRNA expression with real-time PCR: total RNA was isolated from 1 × 10^6^ P3-MSCs or P30-MSCs using TRIzol (Invitrogen) according to the manufacturer’s instructions. First-strand complementary DNA was reverse-transcribed using a Reverse Transcriptase MIX Kit (Toyobo, Osaka, Japan). Quantitative real-time PCR was performed in an ABI 7500 Fast Real-Time PCR System (Applied Biosystems, Foster City, CA, USA) using Fast SYBR Green PCR Master Mix (Applied Biosystems). We used the following primer pairs as reported previously [31]: β-actin (forward: 5′-GAAGGTGAAGGTCGGAGTCA-3′, reverse: 5′-GAAGATGGTGATGGGATTTC-3′) and *LGALS3* (forward: 5′-CCAAAGAGGGAATGATGTTGCC-3′, reverse: 5′-TGATTGTACTGCAACAAGTGAGC-3′).

Galectin-3 levels in CM-P3 or CM-P30 were measured as described previously [25]. Briefly, the concentrated CM was analyzed using a Human Galectin-3 Platinum ELISA (enzyme-linked immunosorbent assay) Kit (eBioscience, San Diego, CA, USA) according to the manufacturer’s instructions.

### Galectin-3 knockdown

To knock down galectin-3 expression, P30-MSCs were transfected with predesigned small interfering RNA (siRNA) (Sigma-Aldrich) using Lipofectamine RNAiMAX reagent (Invitrogen). The respective siRNA sequences of MSCs are as follows: galectin-3 siRNA target site (MSC_gal-3▼_): 5′-CAC UUU AAC CCA CGC UUC AdTdT-3′ and 5′-UGA AGC GUG GGU UAA AGU GdTdT-3′; negative control sequences (MSC_NC_): 5′-UUC UCC GAA CGU GUC ACG UTT-3′ and 5′-ACG UGA CAC GUU CGG AGA ATT-3′.

P30-MSCs were seeded in 6-well plates, and then transfected with a final concentration of 40pmol/μl MSC_gal-3▼_ or MSC_NC_ and incubated for 24 h. Following the incubation and supernatant collection, total RNA or proteins were prepared and quantified as described above. Galectin-3 was quantified using the Human Galectin-3 Platinum ELISA Kit.

Moreover, the addition of recombinant galectin-3 (R&D Systems, Minneapolis, MN, USA) with a final concentration of 50-100nmol/ml to the CM of the galectin-3 knockdown MSCs, and the proliferation of LoVo cells were analyzed.

### Western blotting for mitogen-activated protein kinase (MAPK)

LoVo cells were incubated with CM of MSCs treated with siRNA in the presence or absence of U0126, the specific inhibitor of MEK1/MEK2. Proteins were extracted from LoVo cells using RIPA buffer, separated by 10 % SDS-PAGE, and transferred to Immobilon PVDF membranes (Millipore). The membranes were blocked with PBS containing 5 % non-fat dry milk and 0.1 % Tween 20 overnight. After washing, the membranes were incubated with antibodies against phosphorylated extracellular signal–regulated kinase 1/2 (pERK1/2) and total ERK1/2 (tERK1/2) (Cell Signaling Technology, Inc., Danvers, MA, USA), incubated with HRP-conjugated secondary antibody and visualized with SuperSignal West Pico Chemiluminescent Substrate (Millipore).

### Statistical analysis

The data are presented as the mean ± standard deviation from ≥3 experiments analyzed using SPSS 17.0 statistical software (IBM, New York, NY, USA). Analysis of significance between two groups was performed with an unpaired 2-tailed Student *t*-test; 1-way analysis of variance was used for multiple comparisons. Differences were considered significant when *P* < 0.05.

## Abbreviations

ARF: ADP ribosylation factor
CM: Conditioned media
DMEM: Dulbecco’s modified Eagle’s medium
ELISA: Enzyme-linked immunosorbent assay
ERK: Extracellular signal-regulated kinase
FBS: Fetal bovine serum
HLA-DR: Human leukocyte antigen-DR
HRP: Horseradish peroxidase
INK4a: Cyclin-dependent kinase inhibitor 2A
MAPK: Mitogen-activated protein kinase
MSCs: Mesenchymal stem cells
P3: Passage 3
P30: Passage 30
PBS: Phosphate-buffered saline
PVDF: Polyvinyl difluoride
Q-PCR: Quantitative polymerase chain reaction
RIPA: Radioimmunoprecipitation assay
SA-β-Gal: Senescence-associated β-galactosidase
SASP: Senescence-associated secretory phenotype
SDS-PAGE: Sodium dodecyl sulfate–polyacrylamide gel electrophoresis
siRNA: Small interfering RNA
AD-MSCs: Adipose derived mesenchymal stem cells
